# Computer-Aided Chemistry at Surrey — the way ahead

**DOI:** 10.1155/S1463924689000106

**Published:** 1989

**Authors:** G. J. Buist, B. Howlin, J. R. Jones, D. C. Povey

**Affiliations:** Department of Chemistry University of Surrey Surrey Guildford GU2 5XH UK

## Abstract

This article describes the objectives and rationale of the degree course in Computer-Aided Chemistry at the University of Surrey. The course, which is the first of its kind, represents a sharp break with the past in that industry was intimately involved in the early stages of the planning as well as providing subsequent support; furthermore, much of the teaching is done via a workshop approach. The course aims to produce high-calibre chemists, particularly of the analytical kind, with a firm foundation in computing and having benefited from the close collaboration and cooperation of industry.


Journal of Automatic Chemistry Vol. 11, No. 1 (January–February 1989), pp. 42–43

### Computer-Aided Chemistry at Surrey – the way ahead

**G. J. Buist, B. Howlin, J. R. Jones and D. C. Povey**
*Department of Chemistry, University of Surrey, Guildford, Surrey GU2 5XH, UK*

*This article describes the objectives and rationale of the degree course in Computer-Aided Chemistry at the University of Surrey. The course, which is the first of its kind, represents a sharp break with the past in that industry was intimately involved in the early stages of the planning as well as providing subsequent support; furthermore, much of the teaching is done via a workshop approach. The course aims to produce high-calibre chemists, particularly of the analytical kind, with a firm foundation in computing and having benefited from the close collaboration and cooperation of industry.*

### Introduction

The first 10 years of the *Journal of Automatic Chemistry* [1] have demonstrated the enormous impact of microprocessors in the field of analytical chemistry. This has meant that for the analytical chemist, there is an immediate requirement to become conversant with computer programming, interfacing and data-base technology. These techniques greatly enhance the use that can be made of the analytical data, whilst also boosting the speed of its acquisition.

This growth has to be viewed alongside the comparatively poor representation of analytical chemistry in the UK's higher education system. Consequently, there is a demand in industry for well-trained analysts with good computing skills, which at the current time far exceeds the supply. One of the aims of the Computer-Aided Chemistry degree course at Surrey [2,3] is to redress the balance and to provide its graduates with excellent employment prospects.

### The course

The initial planning of the degree course was undertaken in conjunction with the course advisers:

Mr A. Honeybone, formerly of the Department of Trade and Industry and now Glaxo.
Dr J. G. Vinter, Head of the Department of Molecular Graphics and Computational Chemistry, Smith Kline & French.
Dr S. Ramdas, Analytical Division, British Petroleum.
Dr A. P. Johnson, Director of the Wolfson Unit for Computer-Aided Design of Organic Synthesis, University of Leeds.
Dr S. Neidle, Cancer Research Campaign Biomolecular Structure Unit at Sutton.

As Dr Vinter perceptively mentioned at the time 'The next generation of computer technology will be derived from the scientist versed in computers and not the computer expert who takes his guidance from the scientist'. Consequently, the chemistry content of the course could not be diluted from the level offered in the single honours degree course in chemistry. This approach represents a radical departure from the already existing joint honours degree courses in chemistry and computer science available at several UK universities. It also represents a unique opportunity of integrating the students computing skills with his/her knowledge of chemistry. The course is therefore more difficult and time consuming than an ordinary chemistry degree course and this explains why the 'A' level entry grades, typically a B, a B and a C, are high. In this way a pool of highly motivated and well-qualified students are able to benefit from our philosophy of 'hands-on' experience and a workshop approach to the computing content.

Collaboration with industry is an integral part of the course. This is reflected in two ways – industrialists lecture on the course and the students spend their third year in industry working on some aspects of computer-aided chemistry. This collaboration can be extended to the final year of the course where the student undertakes a lengthy project which may contain elements of the work undertaken during the industrial year. We believe that these circumstances will ensure that the course provides products which will be in much demand by industry.

Setting up the course has cost in the region of £400K. Whilst the university has been very supportive the unfavourable financial climate has meant that industry has borne the brunt of the requirement. Fortunately, Perkin-Elmer were very supportive of the course from its inception and provided both equipment and software, along with an annual scholarship for a student, as did IBM. More recently Hewlett-Packard and Glaxo have joined forces in an imaginative way in sponsoring the course for a five-year period – the former has provided equipment and hardware, whilst Glaxo cover the cost of installation and maintenance as well as providing relevant technical training. Software under favourable terms has been provided by Chemical Design Ltd and ECS Chemical Systems Ltd. The Department is, therefore, fortunate in having a laboratory specifically set aside for teaching the course. Each student has access to his/her own microcomputer, as well as to departmental minicomputers and the university mainframe computer.

### Course content

In the first two years of the course the lecture content is the same as that offered to students on the single honours degree course, but approximately half the practical time

0142–0453/89 $3.00 © 1989 Taylor & Francis Ltd.

is devoted to computing. This means that they acquire a working knowledge of computer architecture coupled with program design in the first year whilst a major part of the second year is spent studying the principles of interfacing equipment to microprocessors. The students also gain experience in operating systems, an introduction to graphics and some of the newer languages so that they are well prepared for the transition to industry in their third year.

Final year students take two options (one major one minor) that are common to the single honours chemistry degree course, for example organometallic and complex chemistry, and a similar number relating to the computer-aided chemistry. One of these is concerned with the application of microcomputers to chromatography, spectroscopy and other analytical techniques, and the other directed towards molecular graphics and computer-aided synthesis. In addition, the project which they undertake provides experience in developing problem-solving skills.

### Conclusion

During the next decade, which will usher in the new era of the super-computer and thereby enhance the importance of computer-aided chemistry, the number of undergraduates studying chemistry is expected to show a sharp decline whilst industry's requirements for high-quality chemists is likely to increase. Could it not be that the present course development with its emphasis on close academic–industrial links points the way ahead?

### References

1. Stockwell P. B., *Journal of Automatic Chemistry*, **10** (1988), p. 1.
2. Buist, G. J., Jones, J. R. and Povey, D. C., *Chemistry in Australia*, **53** (1986), p. 266.
3. Buist, G. J., *CADCAM International* (April 1988), p. 63.

### Book review

**Methodology and Clinical Applications of Ion-selective Electrodes, Volume 9. Proceedings of an International Symposium on the Measurement of Blood Electrolytes Organised by the Electrolyte/Blood Gas Division, American Association for Clinical Chemistry and the European Working Group on Ion-selective Electrodes.**

This collection of reports, as presented at the Symposium held at Danvers, Massachusetts (USA) in September 1987 represents an attempt to gather the written record of current thought on some of the critical issues in electrolyte measurement and clinical application.

The following sessions were organised and reports on these are included in this Monograph: Sodium and potassium standardization; Protein effects on ion-selective electrodes; Ionized calcium pre-analytical, analytical and result reporting considerations; Ionized calcium clinical aspects and reference method; Recent advances in potentiometry, amperometry and optical techniques; and ratification of proposed standards and documents.

In compiling these proceedings, the Editors have attempted to adhere to the sequence of the presentation.

The Symposium itself was organized to provide a forum where a consensus on standards as they apply to electrolyte measurement might be reached and then to proceed to the next level of knowledge and clinical applications. The Symposium and this monograph were dedicated to the memory of Harry Weisberg.

Copies of the report can be purchased either from the IFCC Technical Secretariat or from Dr Mary Burritt, Mayo Clinic, Rochester, MN 55905, USA ($10.00 US).

